# Shikonin Induces Glioma Necroptosis, Stemness Decline, and Impedes (Immuno)Proteasome Activity

**DOI:** 10.1155/2024/1348269

**Published:** 2024-03-14

**Authors:** Xianyun Qin, Lu Zhang, Jilan Liu, Yan Lu, Fuyao Zhou, Feng Jin

**Affiliations:** ^1^Medical Research Center, Affiliated Hospital of Jining Medical University, Jining Medical University, Jining, Shandong 272029, China; ^2^Department of Neurosurgery, Affiliated Hospital of Jining Medical University, Jining Medical University, Jining, Shandong 272029, China; ^3^Clinical Laboratory Medicine Department, Jining No.1 People' s Hospital, Shandong First Medical University, Jining, Shandong 272000, China; ^4^Department of Neurosurgery, Qingdao Central Hospital, University of Health and Rehabilitation Sciences and Qingdao Central Hospital Medical Group, Qingdao, Shandong 266042, China

## Abstract

Gliomas, the most prevalent primary intracranial tumors, exhibit notable features such as heightened malignancy, rapid recurrence, and elevated mortality rates. Presently, standard therapeutic approaches yield limited curative outcomes. Shikonin, an extract derived from traditional Chinese medicine, demonstrates notable bioactivity against various tumors, including gliomas. This study elucidates Shikonin's capacity to effectively induce necroptosis in glioma cells, concurrently mitigating glioma stemness, as evidenced by diminished levels of stem cell markers, namely SOX2, CD44, CHI3L1, and CD24. Our findings indicate that Shikonin-induced programed necrosis leads to a downregulation of proteasome activity and a decrease in the expression of immune proteasome subunits PSMB8/9/10 and PSME1/2/3, contributing to the attenuation of stemness in gliomas. This study comprehensively investigates the interplay between (immuno)proteasome dynamics, Shikonin-mediated necroptosis, and the consequential reduction in glioma stemness, both in vitro and in vivo. The discussion extends to the potential of Shikonin as a promising therapeutic agent in the management of gliomas, offering a novel avenue for drug development in this challenging clinical context.

## 1. Introduction

Gliomas represent the most prevalent primary intracranial tumors, constituting 24% of all primary brain and central nervous system neoplasms [1]. The currently employed standard treatment for gliomas proves ineffective, yielding high rates of recurrence, limited survival durations, and unfavorable prognoses for patients [2]. Particularly, glioblastoma multiforme (GBM), comprising 45% of all gliomas, exhibits a disheartening 5-year relative survival rate of merely 5% and a median postoperative survival of 15 months [3]. Despite considerable research efforts, the etiology of gliomas remains elusive. Notably, a subset of glioma stem cells with self-renewal capabilities has been identified, attributing to tumor recurrence and resistance to conventional therapies [4]. These glioma stem cells exhibit enhanced DNA repair activity, rendering them more resistant to DNA damage [5]. Their pivotal role in maintaining the mesenchymal state, crucial for the highly malignant proliferation of gliomas, underscores the clinical significance of exploring novel antitumor drugs targeting glioma stemness [6, 7].

Shikonin, the principal active constituent of shikon, a natural product-derived compound, has gained clinical attention for its diverse therapeutic applications [8]. Manifesting as a flaky crystalline powder with a molecular formula of C_16_H_16_O_5_ and a weight of 288.299, Shikonin exhibits a spectrum of bioactivities, including antibacterial, anti-inflammatory, antiviral, and antitumor properties [9, 10]. Its effectiveness extends to various cancers, such as esophageal [11], breast [12], kidney [13], lung [14], and liver cancers [15]. Recent research by Zhou et al. [16] elucidates Shikonin's induction of reactive oxygen species (ROS) production via the RIP1/RIP3/MLKL pathway and the programed necrosis of glioma cells. Shikonin's antitumor effects on gliomas involve necroptosis mediated by RIP-1 [17] or downregulation of CD147 [18], with MLKL contributing through the promotion of chromatinolysis [19, 20]. However, a comprehensive understanding of Shikonin's mechanisms in gliomas necessitates further in-depth investigations.

The proteasome, a large protein complex orchestrating protein degradation, operates through the ubiquitin-proteasome system, a fundamental eukaryotic cellular mechanism [21]. Malignant tumor proliferation is characterized by heightened protein synthesis and degradation, positioning the proteasome as a potential target for antitumor therapies [22]. The 26S sedimentation coefficient of the proteasome, obtained through density gradient centrifugation, comprises 20S catalytic and 19S regulatory particles [23]. The cylindrical 20S particle consists of four stacked heteroheptameric rings, housing *α* and *β* subunits, with the inner *β* rings featuring active sites—*β*1(PSMB1), *β*2(PSMB2), and *β*5(PSMB5)—forming the catalytically active core [24]. In response to inflammatory cytokines, especially interferon-gamma (IFN-*γ*), cells express immunoproteasome subunits *β*1i (PSMB9), *β*2i (PSMB10), and *β*5i (PSMB8), replacing the constitutive subunits and forming the immunoproteasome [25, 26]. These (immuno)proteasome components play a crucial role in maintaining glioma stem cell characteristics and disease progression. A siRNA screening analysis targeting genes relevant for GBM survival identified that 22% (12/55) were components of the 20S and 26S proteasome subunits [27]. Gliomas exhibit alterations in the ubiquitin–proteasome system, modulating the expression or activity of numerous enzymes and thereby influencing glioma tumorigenesis [28]. Our previous study showed that corilagin promotes apoptosis in glioma U251 cells by inhibiting the expression and activity of both constitutive and immune proteasomes [29].

This study elucidates the antiglioma mechanism of Shikonin, particularly its role in necroptosis and its efficacy in reducing glioma stemness both in vivo and in vitro. Additionally, it reviews the involvement of the (immuno)proteasome in Shikonin's anti-glioma activity and explores the potential of Shikonin as a targeted drug treatment for gliomas.

## 2. Materials and Methods

### 2.1. Chemicals and Reagents

The Shikonin analytical standard, with a purity exceeding 99%, was purchased from Sigma–Aldrich (St. Louis, MO, USA). Necrostatin-1 (analytical standard, HPLC ≥ 98%), Z-VAD-FMK (analytical standard, HPLC ≥ 95%), GSK-872, and Necrosulfonamide were obtained from Med Chem Express. The Proteasome-Glo™ Cell-Based Assays detection kit was purchased from Promega. Additionally, antibodies against PSMB1, PSMB2, PSMB5, PSMB8, PSMB9, PSMB10, PSME1, PSME2, PSME3, CD44, CHI3L1, SOX2, RIP1, RIP3, and MLKL were acquired from Abcam (Cambridge, MA, USA), while anti-CD24, anti-*β*-actin, and goat antimouse and antirabbit IgG secondary antibodies were procured from Abclonal. The Annexin V-APC/7-AAD Apoptosis Detection Kit was obtained from BioLegend. Furthermore, DMEM/high glucose medium, streptomycin, penicillin, and fetal calf serum were sourced from Gibco Hyclone and trypsin from Gibco (California, CA, USA).

### 2.2. Cell Line and Stem-Like Cell Culture

Human glioma cell lines, namely U251, U87, A172, and T98G, were procured from the China Center for Type Culture Collection (CCTCC) in Wuhan, China. The U251, U87, and A172 cells were maintained at 37°C in 5% CO_2_, cultured in a medium comprising DMEM/high glucose and 10% fetal bovine serum, supplemented with penicillin (100 units/ml) and streptomycin (100 *μ*g/ml) as specified. Meanwhile, T98G cells were cultured in MEM with 10% fetal bovine serum, also supplemented with penicillin (100 units/ml) and streptomycin (100 *μ*g/ml). The U87 and T98G stem-like cells were cultured in F12 medium with the addition of EGF (20 ng/ml), bFGF (20 ng/ml), LIF (10 ng/ml), and 1 *⁣*^*∗*^ B27. To ascertain the impact of Shikonin on programed necrosis in glioma cells and stem-like cells, the four cell lines underwent intervention with different concentrations of Shikonin (0, 4, 8, and 12 *μ*M) for 24 or 48 hr. Then, flow cytometry was employed to assess cell death. In order to determine whether Shikonin-induced cell differentiation involved programed necrosis or apoptosis, cells were treated with distinct concentrations of the programed necrosis inhibitors RIP1 inhibitor NEC-1 (0, 50, 75, and 100 *μ*M), RIP3 inhibitor GSK-872 (0, 10, 15, and 20 mM), MLKL inhibitor Necrosulfonamide (0, 50, 75, and 100 *μ*M), and the apoptosis inhibitor Z-VAD-FMK (0, 5, 10, and 15 mM) in the U251 cell line for 24 hr, and then necrosis detection was performed via flow cytometry to evaluate U251 cell mortality.

### 2.3. Necroptosis/Apoptosis Assay by Flow Cytometry

U251, U87, A172, and T98G cells were treated with Shikonin at a series of concentrations (0, 4, 8, and 12 *μ*M) for 24 hr, while stem-like cells were subjected to the treatment for 48 hr. U251 cells underwent coculture with necrosis/apoptosis inhibitors for 24 hr. Subsequently, cell precipitates were collected, and the cells were stained with Annexin V/PI Apoptosis Detection Kit and tested using Beckman's flow cytometry. The acquired data were then analyzed utilizing the CytExpert for dxFlex software.

### 2.4. Western Blot Analysis

Cells, with a density ranging from 50%–80%, were subjected to varying concentrations of Shikonin (0, 4, 8, and 12 *μ*M) and cultured in six-well plates. After 24 hr, protein extraction was performed, and the protein concentration was assessed utilizing the BCA protein assay kit. Subsequently, cells were collected and lysed using the cell lysis buffer RIPA (Invitrogen). The resulting cell lysates underwent separation through standard SDS–PAGE and were then analyzed by western blot. Antibodies targeting RIP1, RIP3, MLKL, CD44, CD24, CHI3L1, SOX2, PSMB1, PSMB2, PSMB5, PSMB8, PSMB9, PSMB10, PSME1, PSME2, PSME3, and *β*-actin were employed in this analysis. For signal development, the Luminata Forte Western HRP Substrate (Millipore) was utilized. The gray value of each band was individually calculated using Image J software, and the expression level of the target protein was verified by the ratio of the gray value of the target protein to the gray value of Actin (Act). This ratio represents the relative expression of the target protein. The experiment was replicated three times for validation.

### 2.5. Assay for Proteasome Activity

The analysis of 26S proteasome activity was conducted utilizing a previously published method employing the Promega Proteasome Activity Assay Kit [30]. U251 cells, cocultured with varying concentrations of Shikonin (0, 4, 8, and 12 *μ*M) for 24 hr, were subsequently collected. Then, 10,000 cells were seeded in DMEM/high glucose medium and incubated with a detection reagent containing fluorescent substrates with caspase-like protease, trypsin-like enzyme, and chymotrypsin-like activity at 37°C for 30 min. The trypsin-like activity assessment was performed in the presence of a proteasome inhibitor, epoxomycin (5 *μ*M). The fluorescence intensity was quantified with excitation at 380 nm and emission at 460 nm.

### 2.6. RNA-Seq

U251 cells were cultured separately in the vehicle and Shikonin (10 *μ*M) for 12 hr, each with three replicates. Subsequently, transcriptome sequencing was conducted by Berry Genomics Company (Beijing, China).

### 2.7. Nude Mice Tumor Xenograft

Four-week-old athymic BALB/c nude mice were procured from Beijing Sibeifu Biotechnology Co., Ltd. (Beijing, China). The study received approval from the Ethics Committee of the Affiliated Hospital of Jining Medical University (Jining, China) (2021C180). To establish a subcutaneous xenograft model, 1 × 10^6^ logarithmically growing U251 cells in 100 ml PBS were subcutaneously injected into the right flank of each mouse. Therapeutic interventions commenced when the tumor attained a volume of approximately 40–80 mm^3^ after 7 days. Then, tumor-bearing mice were randomly divided into a control group (*n* = 8) and an experimental group (*n* = 10). The mice were administered intraperitoneal injections of vehicle or 2 mg/kg Shikonin once every 2 days for a total of four doses. On the day following the last treatment, the mice were euthanized under isoflurane anesthesia, and tumor tissues were excised. The extracted tumor tissues were fixed with 4% PFA, embedded in paraffin, and sectioned into 5 *μ*m-thick slices for subsequent analysis via immunohistochemistry (IHC) staining.

### 2.8. IHC

The xenografted tumors were fixed in 4% paraformaldehyde, embedded in paraffin, and sectioned at a thickness of 5 *μ*m. The paraffin sections were deparaffinized in xylene, rehydrated through graded alcohols, quenched for 10 min with 3% hydrogen peroxide, and rinsed in PBS (pH 7.6). For antigen retrieval, tissue sections underwent boiling in citrate buffer (pH 6.0) for 20 min in a water bath at 95°C. Following a 30 min treatment with 3% goat serum in PBS, the sections were incubated with the primary antibody (1 : 100) at 37°C for 1 hr, followed by the application of the secondary antibody (for 15 min), DAB (resulting in brown staining), and Meyer's hematoxylin (for background staining). In brief, primary antibodies against RIP1, RIP3, MLKL, SOX2, CD44, CHI3L1, CD24, PSMB1, PSMB2, PSMB5, PSMB8, PSMB9, PSMB10, PSME1, PSME2, and PSME3 were used at a dilution of 1 : 100. All sections were then processed using an ABC Elite kit (Vector Laboratories, Burlingame, CA, USA) in accordance with the manufacturer's protocol. The sections were then incubated for 10 min in a DAB substrate solution containing 0.01% hydrogen peroxide. Finally, imaging of all sections was conducted using an Olympus IX71 microscope (Tokyo, Japan).

### 2.9. Statistical Analysis

Each experiment underwent repetition in triplicate. All data are expressed as mean ± SEM. Statistical comparisons involving three or more groups were conducted through one-way ANOVA, followed by the Least-Significant-Difference test, employing SPSS version 13.0 (SPSS Inc., Chicago, IL). *p* < 0.05 was considered statistically significant.

## 3. Results

### 3.1. Shikonin Predominantly Promoted Glioma Cell Lines (U251/U87/A172/T98G) Cell Death

**Figure 1:**
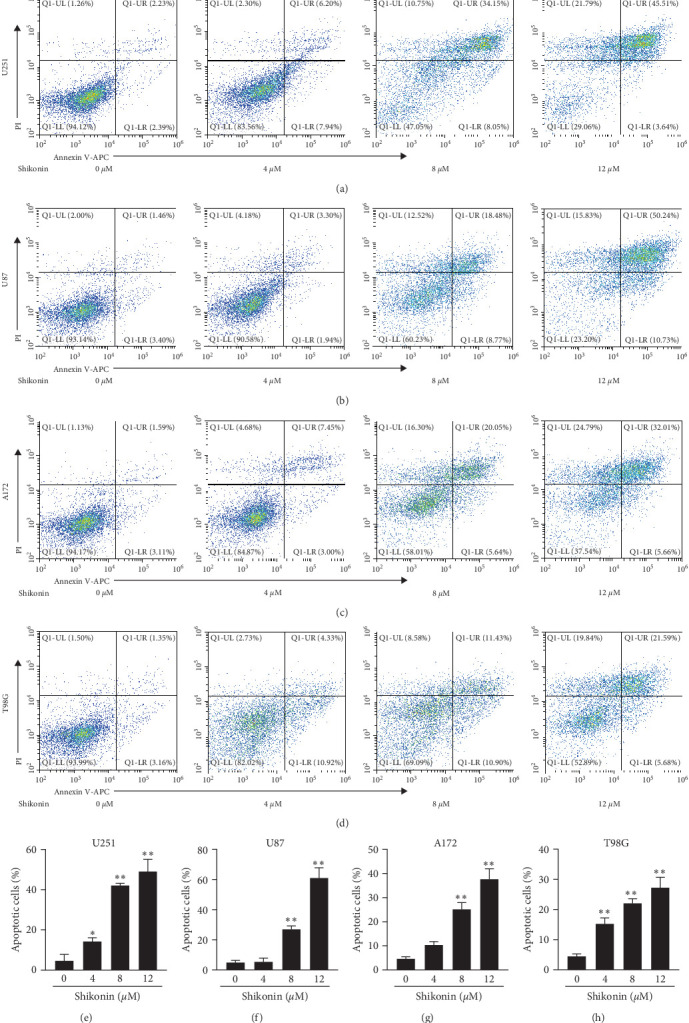
Shikonin predominantly promoted glioma cell lines (U251/U87/A172/T98G) cell death. Images of annexin V/PI doubling staining of (a) U251, (b) U87, (c) A172, and (d) T98G cells treated with increasing concentrations of Shikonin (0, 4, 8, and 12 *μ*M) for 24 hr and then subjected to flow cytometry. The ratios of apoptotic cells (Annexin V^+^/PI^+^ and Annexin V^+^/PI^−^) of (e) U251, (f) U87, (g) A172, and (h) T98G were calculated. Data are expressed as mean ± SEMs (*n* = 5 per group). *⁣*^*∗*^(*p* < 0.05)*⁣*^*∗∗*^(*p* < 0.01) versus untreated cells.

First, we measured the apoptotic rates of glioma cells after Shikonin interventions in glioma cell lines U251/T98G/U87/A172 by using an annexin V/PI staining kit. The results revealed that cell apoptosis rates significantly increased with Shikonin treatment (0, 4, 8, and 12 *μ*M) for 24 hr (Figure 1(a)−1(d)). After 12 *μ*M Shikonin treatment for 24 hr, the ratios of apoptotic cells considerably increased to 49.15%, 60.97%, 37.67%, and 27.27%, respectively (Figure 1(e)–1(h)). Taken together, these results suggested that Shikonin effectively promotes cell death in glioma.

### 3.2. Shikonin Predominantly Promoted U87 and T98G Stem-Like Cells Death

**Figure 2:**
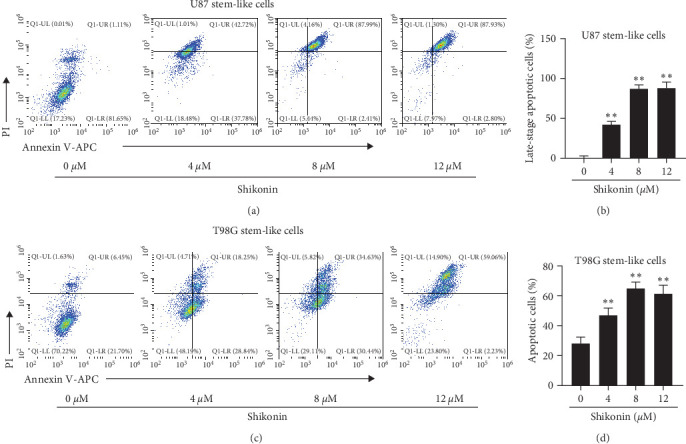
Shikonin predominantly promoted U87 and T98G stem-like cells death. Images of annexin V/PI doubling staining of (a) U87 stem-like cells and (b) T98G stem-like cells treated with increasing concentrations of Shikonin (0, 4, 8, and 12 *μ*M) for 48 hr and then subjected to flow cytometry. The apoptotic cell ratios of (c) U87 stem-like cells (Annexin V^+^/PI^+^) and (d) T98G stem-like cells (Annexin V^+^/PI^+^ and Annexin V^+^/PI^−^) were calculated. Data are expressed as mean ± SEMs (*n* = 5 per group). *⁣*^*∗*^(*p* < 0.05)*⁣*^*∗∗*^(*p* < 0.01) versus untreated cells.

Next, we measured the apoptotic rates of U87 and T98G stem-like cells after Shikonin interventions (0, 4, 8, and 12 *μ*M) for 48 hr. The results revealed that cell apoptosis rates significantly increased with Shikonin treatment (Figures 2(a) and 2(b)). The early apoptotic cell rates of U87 stem-like cells treated with 0, 4, 8, and 12 *μ*M were 1.11%, 42.72%, 87.99, and 87.93, respectively (Figure 2(c)). Apoptotic cell rates (early apoptosis and late apoptosis) of T98G stem-like cells were 28.15%, 47.09%, 65.07%, and 61.29%, respectively (Figure 2(d)). Taken together, these results suggested that Shikonin effectively promotes cell death in stem-like glioma cells.

### 3.3. Necroptosis Inhibitors Can Significantly Reduce Shikonin-Induced Glioma Cell Death

**Figure 3:**
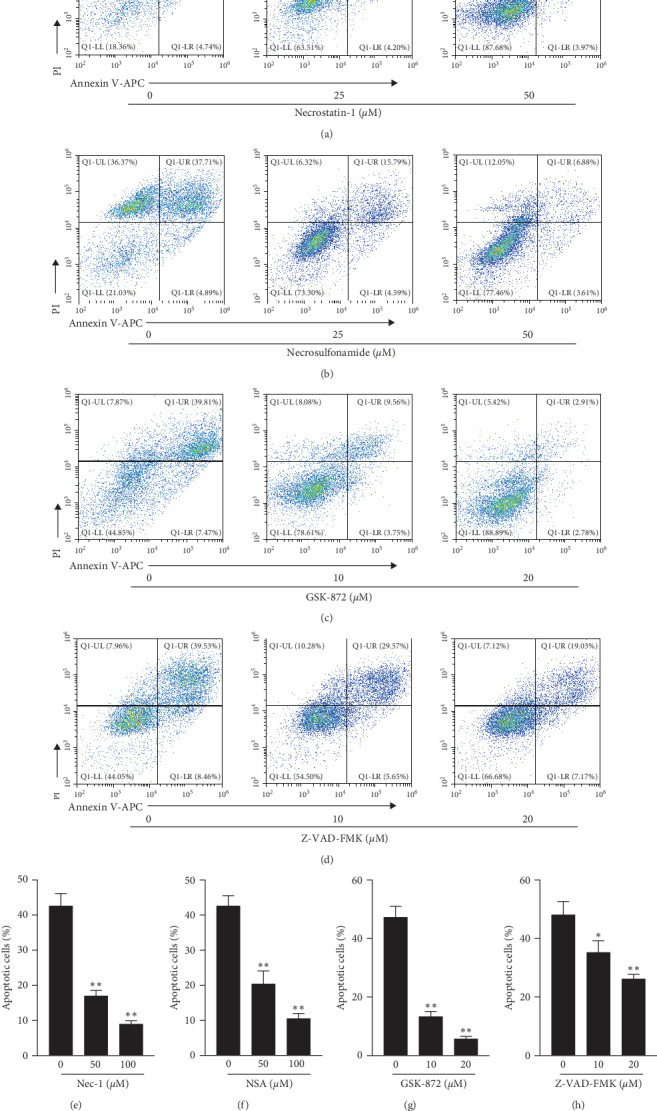
Necroptosis inhibitors can significantly reduce Shikonin-induced U251 cell death. Images of Annexin V/PI doubling staining of U251 increasing concentrations of (a) necrostatin-1(O, 25, and 50 *μ*M), (b) necrosulfomamide (O, 25, 50 *μ*M), (c) GSK-872 (O, 10, and 20 *μ*M), (d) Z-VAD-FMK (O, 10, and 20 *μ*M) cells treated with Shikonin 12 *μ*M for 24 hr and then subjected to flow cytometry. The ratios of apoptotic cells (Annexin V^+^/PI^+^ and Annexin V^+^/PI−) of (e) necrostatin-1, (f) necrosulfomamide, (g) GSK-872, and (h) Z-VAD-FMK were calculated. Data are expressed as mean ± SEMs (*n* = 5 per group). *⁣*^*∗*^(*p* < 0.05)*⁣*^*∗∗*^(*p* < 0.01) versus untreated cells.

Next, we used necrosis/apoptosis inhibitors before Shikonin interventions to treat glioma cells to determine the causative role of Shikonin regulating cell necroptosis or apoptosis. Necroptosis RIP1 inhibitor necrostatin-1 (O, 25, 50 *μ*M), RIP3 inhibitor necrosulfomamide (O, 25, and 50 *μ*M), MLKL inhibitor GSK-872 (O, 10, and 20 *μ*M), and apoptosis inhibitor Z-VAD-FMK (O, 10, and 20 *μ*M) were added to U251 cells 30 min before the addition of Shikonin (12 *μ*M) for 24 hr. Annexin V/PI staining of the above cells and then subjected to flow cytometry. Results showed that the effects of the inhibitors were dose-dependent; lethal rates of U251 were significantly decreased from 42.63% to 8.96% (Nec-1) (Figures 3(a) and 3(e)), 42.6% to 10.49% (necrosulfomamide) (Figures 3(b) and 3(f)), 47.28% to 5.69% (GSK 872) (Figures 3(c) and 3(g)), and 47.99% to 26.2% (Z VAD FMK) (Figures 3(d) and 3(h)). We can see that necroptosis inhibitor treatment was more effective in rescuing cell death than apoptosis inhibitor, and we conclude that Shikonin induced glioma cell death mainly through necroptosis.

### 3.4. Shikonin Increased MLKL/RIP3 Expression Levels

**Figure 4:**
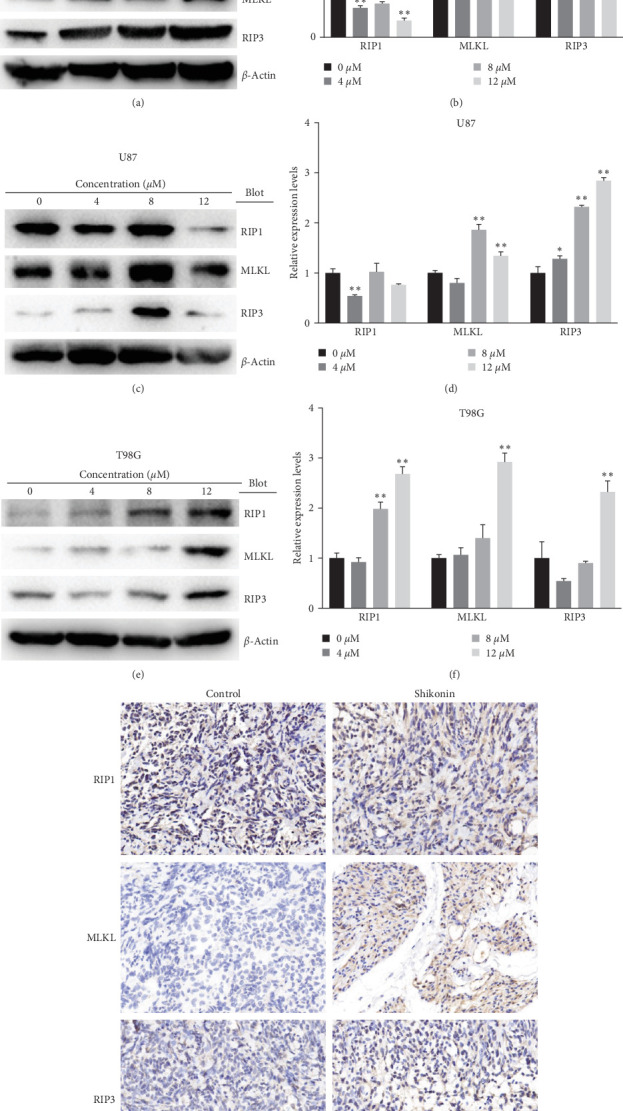
Shikonin increased MLKL/RIP3 expression levels. RIP1/MLKL/RIP3 expression levels of (a) U251, (c) U87, and (e) T98G cells, which were treated with increasing concentrations of Shikonin (0, 4, 8, and 12 *μ*M) for 24 hr, were detected by western blot analysis. The quantification of RIP1/MLKL/RIP3 protein levels of (b) U251, (d) U87, and (f) T98G cells was performed. (g) Immunohistochemical staining of RIP1/MLKL/RIP3 of Shikonin-treated glioma subcutaneous implantation model. Data are expressed as mean ± SEMs (*n* = 3 per group). *⁣*^*∗*^(*p* < 0.05)*⁣*^*∗∗*^(*p* < 0.01) versus untreated cells.

To further explore the effect of Shikonin on necroptosis of glioma cells, we detected necroptosis-related proteins RIP1, RIP3, and MLKL expression levels of U251, U87, T98G cells treated with Shikonin (0, 4, 8, and 12 mM) for 24 hr. The protein levels of the RIP1/MLKL/RIP3 (Figure 4(a)) were detected through western blotting. Shikonin treatment significantly increased RIP1MLKL/RIP3 in T98G, only increased MLKL, RIP3 in U251 and U87(Figure 4(a)−4(f)). Meanwhile, we established a glioma subcutaneously transplanted tumor model. Immunohistochemical staining of RIP1/MLKL/RIP3 of nude mouse subcutaneous tumor model shows the same result (Figure 4(g)).

### 3.5. Shikonin Decreased Glioma Stemness Markers and TAM Markers

**Figure 5:**
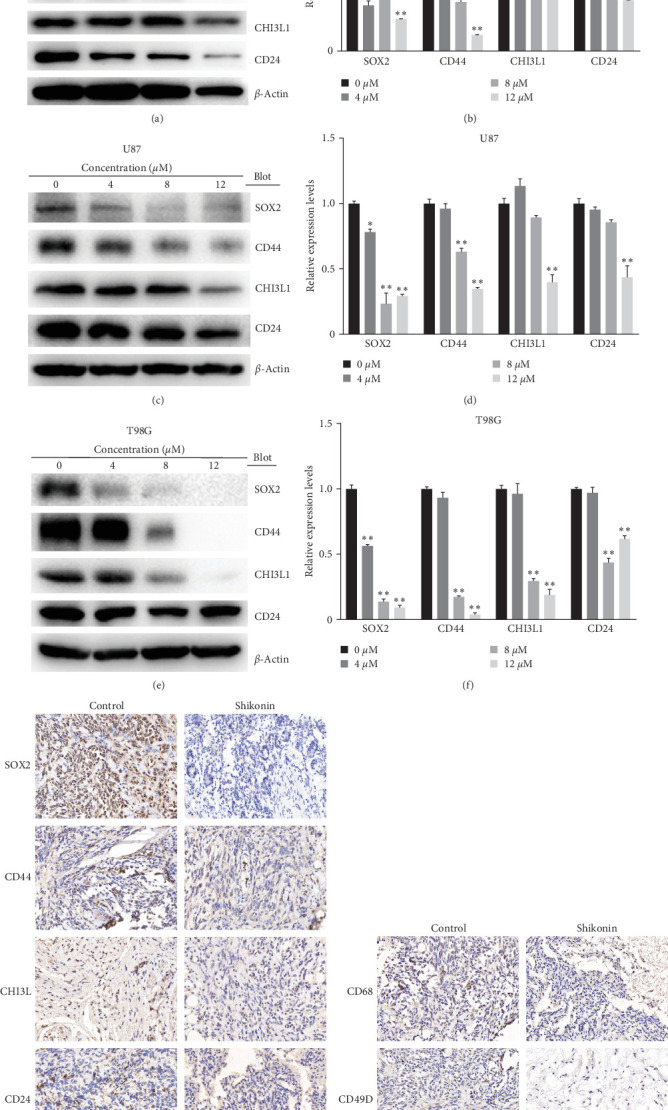
Shikonin decreased glioma stemness markers and TAM markers. SOX2/CD44/CHI3L1/CD24 protein levels of (a) U251, (c) U87, and (e) T98G cells, which were treated with increasing concentrations of Shikonin (0, 4, 8, and 12 *μ*M) for 24 hr, were detected by western blot analysis. The quantification of SOX2/CD44/CHI3L1/CD24 protein levels of (b) U251, (d) U87, and (f) T98G cells were performed. Immunohistochemical staining of (g) SOX2/CD44/CHI3L1/CD24 and (h) CD68/CD49D of Shikonin-treated glioma subcutaneous implantation model. Data are expressed as mean ± SEMs (*n* = 3 per group). *⁣*^*∗*^(*p* < 0.05)*⁣*^*∗∗*^(*p* < 0.01) versus untreated cells.

We also investigated the effect of Shikonin on glioma stemness and TAM (tumor-associated macrophage) infiltration in vivo and in vitro. We treated U251, U87, and T98G cells with Shikonin (0, 4, 8, 12 *μ*M) for 24 hr. The protein levels of stemness markers SOX2, CD44, CHI3L1, CD24, and TAM markers of CD68, CD49D were detected through western blotting (Figure 5(a)−5(f)) and IHC (Figures 5(g) and 5(h)). Results showed that Shikonin treatment significantly reduced the protein levels of SOX2, CD44, CHI3L1, and CD24 in vitro (Figure 5(a)–5(f)). Immunohistochemical staining of nude mouse subcutaneous tumor model of SOX2, CD44, CHI3L1, and CD24 in vivo shows the same result (Figure 5(g)), and IHC staining of TAM markers CD68 had no obvious change (Figure 5(h)). This study also showed that Shikonin reduced the expression of CD49D (Figure 5(h)), which is a distinguishing marker between microglia and BMDM (bone marrow-derived macrophages) of TAM [31].

### 3.6. Shikonin Decreased Proteasome Activities

**Figure 6:**
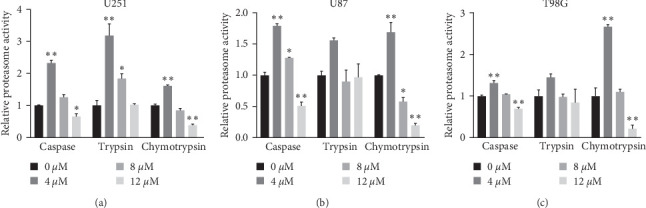
Shikonin reduced proteasome activities. U251, U87, and T98G cells were treated with increasing concentrations of Shikonin (0, 4, 8, and 12 *μ*M) for 24 hr. Caspase-like, trypsin-like, and chymotrypsin-like proteasome activities of (a) U251, (b) U87, and (c) T98G cells were measured by fluorogenic substrates. Data are expressed as mean ± SEMs (*n* = 3 per group). *⁣*^*∗*^(*p* < 0.05)*⁣*^*∗∗*^(*p* < 0.01) versus untreated cells.

Proteasome activities of U251, U87, and T98G cells treated with Shikonin of different concentrations (0, 4, 8, and 12 *μ*M) at 24 hr, including caspase-like, trypsin-like, and chymotrypsin-like activities were measured using fluorescent-labeled peptides. At 4 *μ*M, Shikonin treatment markedly increased caspase-like, trypsin-like and chymotrypsin-like activities of U251 (Figure 6(a)); at 8 *μ*M, the activities had no much changes (Figure 6(a)); at 12 *μ*M, caspase-like, chymotrypsin-like activities remarkably decreased, and trypsin-like activities had no much changes (Figure 6(a)). In U87 and T98G cells, there was no significant change in trypsin-like activity, while caspase-like and chymotrypsin-like activities decreased significantly after 24 hr (Figures 6(b) and 6(c)). These results suggested that Shikonin can decrease the proteasome activities in glioma cells.

### 3.7. Shikonin Increased the Levels of Ubiquitinated Proteins

**Figure 7:**
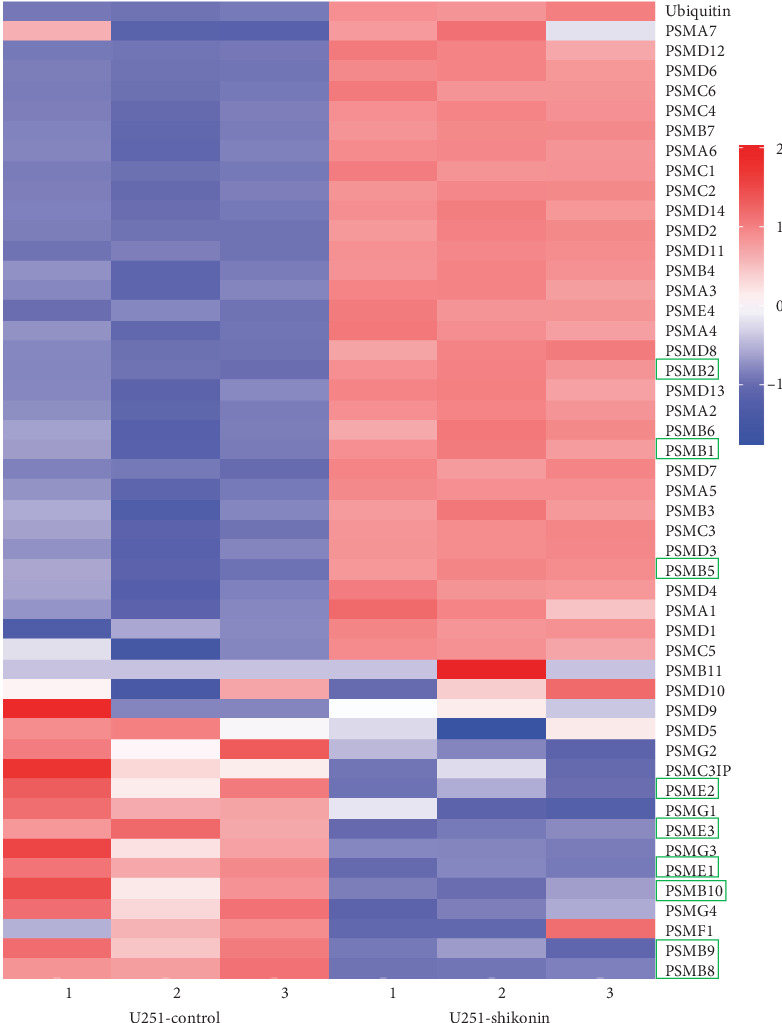
Heatmap showing transcriptomic sequencing results of the effect of Shikonin on the expression levels of ubiquitinated proteins and proteasome subunits. U251 cells were treated with Shikonin for 24 hr; Shikonin decreased the expression levels of immunoproteasome catalytic subunits and increased ubiquited proteins. The green box highlights that the expression of the constitutive proteasome subunits PSMB1, PSMB2, and PSMB5 was increased, while the expression of the immunoproteasome subunits PSMB8, PSMB9, PSMB10, PSME1, PSME2, and PSME3 was decreased.

**Figure 8:**
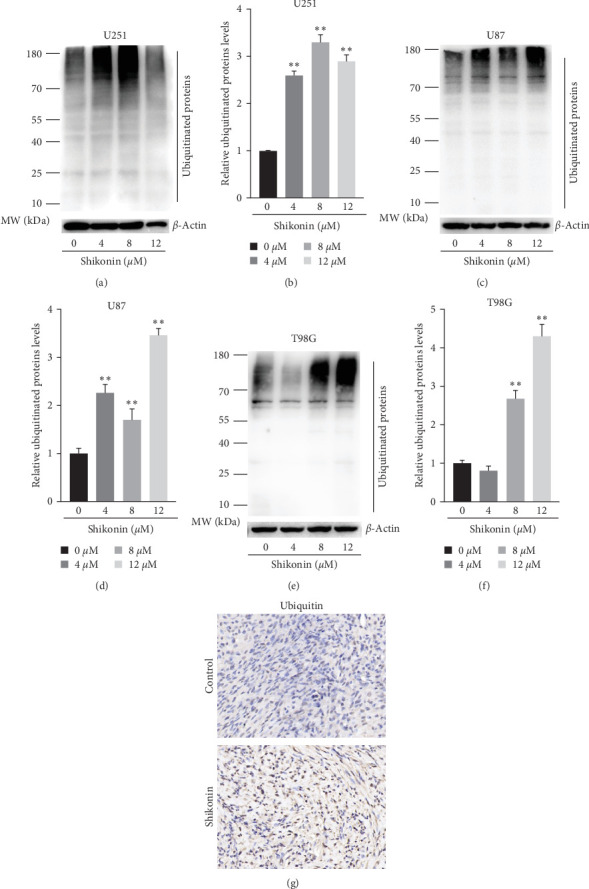
Shikonin increased the levels of poly-ubiquitinated proteins. Ubiquitin expression levels of (a) U251, (c) U87, and (e) T98G cells, which were treated with increasing concentrations of Shikonin (0, 4, 8, and 12 *μ*M) for 24 hr, were detected by western blot analysis. The quantification of ubiquitin protein levels of (b) U251, (d) U87, and (f) T98G cells was performed. (g) Immunohistochemical staining of ubiquitin of Shikonin-treated glioma subcutaneous implantation model. Data are expressed as mean ± SEMs (*n* = 3 per group). *⁣*^*∗*^(*p* < 0.05)*⁣*^*∗∗*^(*p* < 0.01) versus untreated cells.

Transcriptome sequencing was conducted by U251, U87, and T98G cells treated with Shikonin (10 *μ*M) for 12 hr. We analyzed the expression levels of ubiquitin and proteasome subunits of transcriptome sequencing results. Heatmap showed that Shikonin increased ubiquitin and decreased the expression levels of immuno-proteasome catalytic subunits (Figure 7). The protein levels of ubiquitin were detected through western blot and IHC (Figure 8). Western blot showed that Shikonin treatment significantly reduced the protein levels of ubiquitin in U251, U87, and T98G cells (Figure 8(a)−8(f)). Immunohistochemical staining of ubiquitin in vivo shows the same result (Figure 8(g)). Both in vivo and in vitro experiments confirmed that with the increase of Shikonin concentration, the expression level of ubiquitinated protein gradually increased.

### 3.8. Shikonin Decreased the Protein Levels of Immnoproteasome Subunits

**Figure 9:**
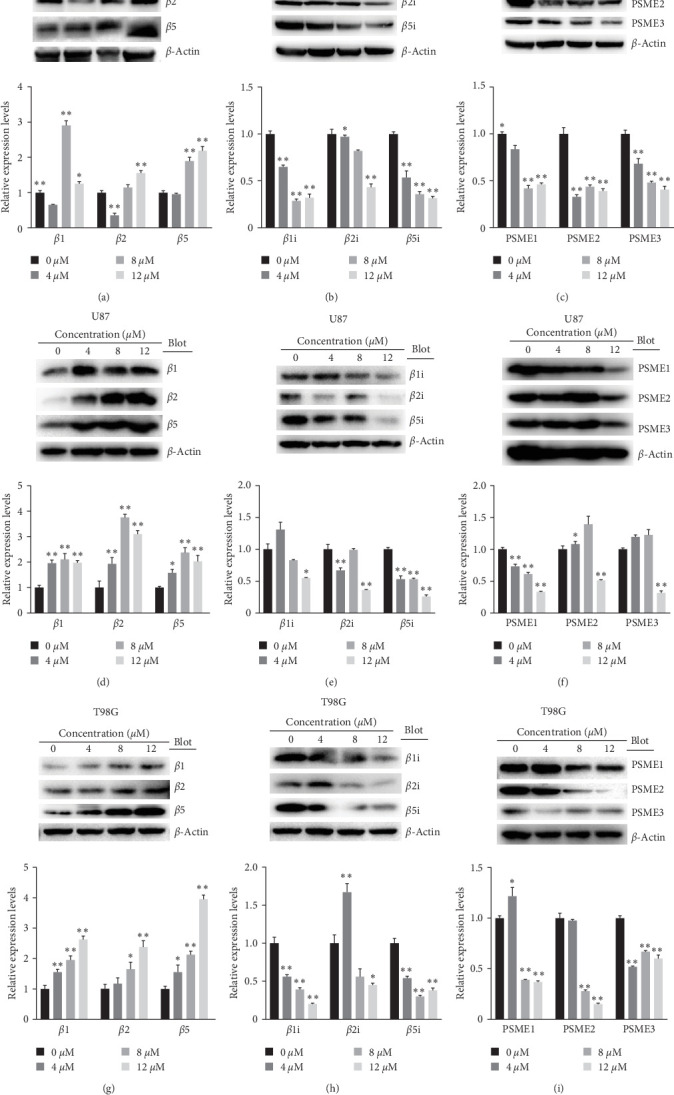
Shikonin increased constitutive proteasome subunits of *β*1, *β*2, and *β*5 and decreased the expression levels of immunoproteasome catalytic subunits of *β*1i, *β*2i, *β*5i, PSME1, PSME2, and PSME3. U251, U87, and T98G cells were treated, increasing with concentrations of Shikonin (0, 4, 8, and 12 *μ*M) for 24 hr. The protein levels of *β*1/*β*2/*β*5 of (a) U251, (d) U87, and (g) T98G cells, *β*1i/*β*2i/*β*5i of (b) U251, (e) U87, and (h) T98G cells, and PSME1/PSME2/PSME3 of (c) U251, (f) U87, and (i) T98G cells were detected by western blot analysis and the quantification was also performed. Data are expressed as mean ± SEM (*n* = 3 per group). *⁣*^*∗*^(*p* < 0.05)*⁣*^*∗∗*^(*p* < 0.01) versus untreated cells.

**Figure 10:**
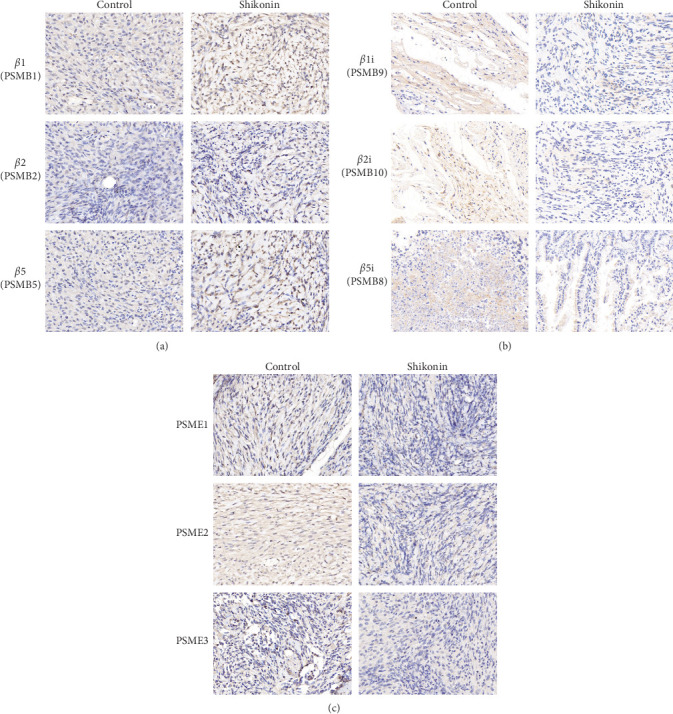
Shikonin increased *β*1/*β*2/*β*5 and decreased *β*1i/*β*2i/*β*5i, PSME1/PSME2/PSME3 expression levels. Immunohistochemical staining of (a) *β*1/*β*2/*β*5, (b) *β*1i/*β*2i/*β*5i, and (c) PSME1/PSME2/PSME3 of Shikonin-treated glioma subcutaneous implantation model.

The transcriptomic heatmap of Figure 7 showed that Shikonin increased ubiquited proteins and decreased the expression levels of immuno-proteasome catalytic subunits. The green box highlights that the expression of the constitutive proteasome subunits PSMB1, PSMB2, and PSMB5 is increased, while the expression of the immunoproteasome subunits PSMB8, PSMB9, PSMB10, PSME1, PSME2, and PSME3 is decreased. To further validate this sequencing result, the protein levels of the constitutive subunits (i.e., PSMB1, PSMB2, and PSMB5) and inducible proteasome catalytic subunits (i.e., PSMB8, PSMB9, PSMB10, PSME1, PSME2, and PSME3) in U251 (Figure 9(a)−9(c)), U87 (Figure 9(d)−9(f)), and T98G (Figure 9(g)–9(i)) were detected through Western blotting and IHC (Figure 10). Shikonin treatment significantly increased the protein levels of constitutive subunits (PSMB1, PSMB2, and PSMB5) but reduced those of the immunosubunits subunit (i.e., PSMB8, PSMB9, PSMB10, PSME1, PSME2, and PSME3; Figures 9 and 10), indicating that the Shikonin-induced decrease in proteasome activity in glioma is mainly mediated by decreased expression of the immunoproteasome.

## 4. Discussion

We observed an upregulation of programed necrosis-related proteins MLKL/RIPK3 and a concomitant downregulation of RIPK1 expression in glioma cell lines U251, U87, A172, and T98G, as well as in an in vivo subcutaneous tumor model, following Shikonin interventions. Glioma cells cultured in a Shikonin-supplemented medium (12 *μ*M) with specific inhibitors Nec-1/NSA/GSK-872 for 24 hr exhibited a decreased programed necrosis rate. This suggests the involvement of the RIP1-RIP3-MLKL signaling pathway in programed necrosis, potentially contributing to Shikonin-induced glioma cell death. Additionally, Shikonin treatments resulted in diminished levels of glioma stem cell markers (SOX2, CD44, CHI3L1, and CD24) and the BMDM-derived TAM marker CD49D in glioma cells and subcutaneous animal models. Furthermore, Shikonin interventions led to alterations in the expression of immunoproteasome subunits. Specifically, PSMB8 (*β*5i), PSMB9 (*β*1i), PSMB10 (*β*2i), PSME1, PSME2, and PSME3 expression decreased, while PSMB1 (*β*1), PSMB2 (*β*2), and PSMB5 (*β*5) increased. The proteasome activator subunits (PSME1, PSME2, and PSME3, also known as PA28 *α*/*β*/*γ*) regulated the immune proteasome, replacing the 19S regulator (also known as the 11S regulator). The *α* and *β* subunits combined to form hexameric rings, while six *γ* subunits formed homohexameric rings [32]. Additionally, the proteasome caspase-like, trypsin-like, and chymotrypsin-like activities generally decreased. Therefore, we hypothesize that Shikonin inhibits the immunoproteasome function of the ubiquitin-proteasome system, promoting glioma necroptosis and reducing the malignancy of glioma stem cells.

The formation of the RIP1-RIP3-MLKL complex is a characteristic feature of necroptosis, where MLKL acts as a crucial executor. Ding et al. [19] demonstrated that MLKL promotes necroptosis by regulating chromatin breakdown, facilitating nuclear translocation of AIF, and the formation of *γ*-H2AX. Lu et al. [33] confirmed Shikonin's activation of RIP1 and RIP3 in glioma cells, both in vitro and in vivo, through the elevation of intracellular H_2_O_2_ and associated glycolysis inhibition. RIP1 and RIP3 contribute to Shikonin-induced DNA double-strand breaks in glioma cells by increasing intracellular ROS [16].

The proteasome, a large protein complex usually in the form of a 26S proteasome, comprises a 20S catalytic and two 19S regulatory subunits. The 20S subunit is composed of four coaxial rings—two outer *α* and two inner *β* chain loops. Each *α* and *β* chain consists of seven subunits, forming a barrel-shaped structure [24]. The functional sites for protein degradation are PSMB1 (*β*1), PSMB2 (*β*2), and PSMB5 (*β*5) subunits. During an immune response, especially influenced by cytokines like IFN-*γ*, there is increased expression of *β* subunits, leading to the formation of “immunoproteasomes” [34], assembled from alternative subunits PSMB8 (*β*5i), PSMB9 (*β*1i), and PSMB10 (*β*2i). The ubiquitin-proteasome system controls cellular processes such as the cell cycle, transcription, signaling, and trafficking [21]. Therefore, drugs targeting the proteasome hold promise for novel tumor and disease treatments. Altered proteasome activity can result in excessive degradation of crucial cellular proteins or the accumulation of pathological proteins. Alturki et al. [35] demonstrated that RIP1 is degraded by the proteasome. TRIM25, a negative regulator of programed necrosis of RIP3, promotes polyubiquitination linkage on RIP3 through its loop domain, facilitating the proteasomal degradation of RIP3 and inhibiting cell necrosis. This implies that the RIP1 pathway is mediated by the proteasome.

Our investigations revealed a downregulation of immunoproteasome subunits, namely PSMB8, PSMB9, PSMB10, PSME1, PSME2, and PSME3, both in vivo and in vitro, following Shikonin treatment. Numerous studies have demonstrated the regulatory roles of PSMB8, PSMB9, and PSMB10 in glioma proliferation, migration [36], angiogenesis [37], inflammatory response [38], and hypoxia-related tumor characteristics [39]. Additionally, PSME1/2/3 are implicated in MHC class I molecule-mediated antigen presentation [40], and their heightened expression is significantly correlated with the staging and prognosis of various tumors, including gastric [40], colorectal [41], clear cell renal cell carcinoma [42], and breast cancer [43]. Guo et al. [40] observed significantly elevated median expression levels of all PSME genes in gastric cancer compared to normal tissues. Guo et al. [44], through qRT-PCR, western blot, Oncomine data mining, and immunohistochemical analyses, demonstrated significant upregulation of PSME3 at both mRNA and protein levels in pancreatic cancer cells and tissues. Increased PSME2 expression was linked to clear cell renal cell carcinoma invasion through regulatory autophagy [42]. Dong et al. [43], in their analysis of 1,039 breast cancer patients from the TCGA database, identified PSME2 as a differentially expressed gene associated with breast cancer prognoses. PSME3 was also overexpressed in relapsed/refractory myelomas and correlated with poor prognoses [45]. Furthermore, it plays a crucial role in antigen processing and serves as a target for cancer immunotherapy [43].

The transcription factor SOX2 is a key regulator of stemness in embryonic and neural stem cells [46]. GBM stem cells exhibit elevated SOX2 levels, which impede astrocyte differentiation and revert the cell to a pluripotent state [47]. SOX2 plays a significant role in the invasive growth of GBM white matter tracts [48]. Benedetti et al. [49] engineered a synthetic repressor named the SOX2 epigenetic silencer (SES), inducing cell death in both glioma cell lines and patient-derived cancer stem cells in vitro and in vivo. In mouse xenografts, SES expression led to robust regression and survival rescue of human tumors through local viral delivery, with no harm to neurons and glial cells. CD44 overexpression is associated with poor prognosis in grade II/III gliomas [50, 51]. CHI3L1, also known as YKL-40, is linked to GBM mesenchymal subtypes [52] and serves as a marker for high-grade glioma [53, 54]. CHI3L1 is strongly associated with immunosuppression in glioma-associated macrophages [55]. CD24 signaling, through Siglec-10 macrophages, is a target for cancer immunotherapy [56] and is related to the mutant-IDH1-dependent chromatin state [57].

Furthermore, we demonstrated that Shikonin diminishes the expression of CD49D in glioma-associated macrophages, a marker distinguishing microglia from BMDM [31], with concurrent enrichment of glioblastoma-infiltrating T-cell populations [58]. CD49D emerged as an independent factor for PFS in 60 GBM patients, as determined by Cox proportional hazards regression analysis, positioning it as a potential GBM biomarker [59].

For the successful clinical application of Shikonin, numerous challenges must be addressed. Shikonin faces difficulty traversing the blood–brain barrier, but Li et al. [60] devised PEG-PLGA nanoparticles coated with lactoferrin to enhance their ability to cross the blood–brain barrier and reach receptor-mediated pathway targets on glioma cells. To mitigate Shikonin's side effects, structural and functional group modifications can be explored. This study aimed to redirect glioma treatment towards traditional Chinese medicine, establishing new targets and approaches theoretically and experimentally.

## Data Availability

The datasets used and/or analyzed during the current study are available from the corresponding author upon reasonable request.
